# Recruitment-to-inflation ratio reflects the impact of peep on dynamic lung strain in a highly recruitable model of ARDS

**DOI:** 10.1186/s13613-024-01343-w

**Published:** 2024-07-04

**Authors:** Francesco Murgolo, Domenico L. Grieco, Savino Spadaro, Nicola Bartolomeo, Rossella di Mussi, Luigi Pisani, Marco Fiorentino, Alberto Maria Crovace, Luca Lacitignola, Francesco Staffieri, Salvatore Grasso

**Affiliations:** 1 Department of Precision-Regenerative Medicine and Jonic Area (DiMePRe-J), Section of Anesthesiology and Intensive Care Medicine, University of Bari “Aldo Moro”, Bari, Italy; 2 Department of Anesthesia, Intensive Care and Emergency, Fondazione Policlinico A. Gemelli IRCCS, Rome, Italy; 3 Department of Anesthesiology and Intensive Care Medicine, Catholic University of the Sacred Heart, Rome, Italy; 4 Department of Translational Medicine, Section of Anesthesiology and Intensive Care Medicine, University of Ferrara, Ferrara, Italy; 5 Interdisciplinary Department of Medicine, University of Bari “Aldo Moro”, Bari, Italy; 6 Nephrology, Dialysis and Transplantation Unit, Department of Precision and Regenerative Medicine and Ionian Area (DiMePRe‐J), University of Bari, Bari, Italy; 7 Department of Veterinary Medicine, University of Sassari, Sassari, Italy; 8 Department of Precision-Regenerative Medicine and Jonic Area (DiMePRe-J), Section of Veterinary Medicine, University of Bari “Aldo Moro”, Bari, Italy; 9 Dipartimento di Medicina di Precisione e Rigenerativa e Area Jonica (DiMePRe-J), Sezione di Anestesiologia e Rianimazione, Ospedale Policlinico, Università Degli Studi “Aldo Moro”, Piazza Giulio Cesare 11, Bari, Italy

**Keywords:** Acute respiratory distress syndrome, Mechanical ventilation, Recruitment-to-inflation ratio, Lung recruitment, Dynamic lung strain

## Abstract

**Background:**

The recruitment-to-inflation ratio (R/I) has been recently proposed to bedside assess response to PEEP. The impact of PEEP on ventilator-induced lung injury depends on the extent of dynamic strain reduction. We hypothesized that R/I may reflect the potential for lung recruitment (i.e. recruitability) and, consequently, estimate the impact of PEEP on dynamic lung strain, both assessed through computed tomography scan.

**Methods:**

Fourteen lung-damaged pigs (lipopolysaccharide infusion) underwent ventilation at low (5 cmH_2_O) and high PEEP (i.e., PEEP generating a plateau pressure of 28–30 cmH_2_O). R/I was measured through a one-breath derecruitment maneuver from high to low PEEP. PEEP-induced changes in dynamic lung strain, difference in nonaerated lung tissue weight (tissue recruitment) and amount of gas entering previously nonaerated lung units (gas recruitment) were assessed through computed tomography scan. Tissue and gas recruitment were normalized to the weight and gas volume of previously ventilated lung areas at low PEEP (normalized-tissue recruitment and normalized-gas recruitment, respectively).

**Results:**

Between high (median [interquartile range] 20 cmH_2_O [18–21]) and low PEEP, median R/I was 1.08 [0.88–1.82], indicating high lung recruitability. Compared to low PEEP, tissue and gas recruitment at high PEEP were 246 g [182–288] and 385 ml [318–668], respectively. R/I was linearly related to normalized-gas recruitment (r = 0.90; [95% CI 0.71 to 0.97) and normalized-tissue recruitment (r = 0.69; [95% CI 0.25 to 0.89]). Dynamic lung strain was 0.37 [0.29–0.44] at high PEEP and 0.59 [0.46–0.80] at low PEEP (p < 0.001). R/I was significantly related to PEEP-induced reduction in dynamic (r = − 0.93; [95% CI − 0.78 to − 0.98]) and global lung strain (r = − 0.57; [95% CI − 0.05 to − 0.84]). No correlation was found between R/I and and PEEP-induced changes in static lung strain (r = 0.34; [95% CI − 0.23 to 0.74]).

**Conclusions:**

In a highly recruitable ARDS model, R/I reflects the potential for lung recruitment and well estimates the extent of PEEP-induced reduction in dynamic lung strain.

**Supplementary Information:**

The online version contains supplementary material available at 10.1186/s13613-024-01343-w.

## Introduction

In acute respiratory distress syndrome (ARDS), mechanical ventilation should sustain gas exchange while minimizing ventilator-induced lung injury (VILI) [1]. Dynamic lung strain, defined as the ratio of tidal volume (V_T_) to functional residual capacity (FRC), is one of the VILI determinants. In healthy pigs submitted to mechanical ventilation, dynamic lung strains higher than 1.5 has been shown to be “lethal” [2]. Less is known about the “critical” threshold in damaged, inhomogeneous lungs as during ARDS [3]. However, studies on patients showed that a dynamic strain of 0.27 may be deemed “high,” as it is associated with a proinflammatory lung response in patients with acute lung injury [4].

Well-established practices strongly emphasize the use of low V_T_ and prone position to homogenize lung aeration [5, 6], while the role of positive end-expiratory pressure (PEEP) is still debated [7, 8]. In highly recruitable patients, PEEP recruits collapsed lung tissue, increasing FRC and decreasing dynamic lung strain [9, 10]. However, in every case, PEEP invariably inflates already aerated lung regions [10–12], increasing static lung strain [13] even in good recruiters. Nevertheless, evidence suggests that dynamic lung strain is more implicated in VILI generation than static lung strain [13–15] and, accordingly, tailoring PEEP to minimize dynamic lung strain is a physiologically straightforward target. In this context, a tool to define the impact of PEEP on dynamic lung strain would be welcome [16]. Computed tomography (CT) is the gold standard to assess lung strain and the impact of PEEP on alveolar recruitment [9, 17] but remains impractical for routine clinical use [18]. This moved clinical research toward the development of bedside strategies to best phenotype patient responses to PEEP in clinical practice.

The recruitment to inflation ratio (R/I) has been recently proposed to assess lung recruitability at the bedside [19]. Through a simplified derecruitment maneuver [20], this new index quantifies lung recruitability by scaling the compliance of recruited tissue (C_REC_) to that of the previously aerated lung at low PEEP [19]. Briefly, by simplifying the original multiple pressure/volume curve technique [21], the R/I discriminates recruiters from non-recruiters by assuming that if C_REC_ at higher PEEP is at least 50% of the respiratory system compliance at lower PEEP (i.e., R/I > 0.5), the balance between PEEP-induced alveolar recruitment and inflation is in favor of recruitment [19].

By predicting and quantifying lung recruitability, the R/I should reflect the impact of PEEP on dynamic lung strain [22]. However, to our knowledge, no study has assessed whether R/I accurately reflects dynamic lung strain lung and recruitability compared with the “gold standard” CT scan method. Therefore, we conducted an experimental study on a high recruitable ARDS model ventilated with two PEEP levels (low and high) in the context of a low-tidal volume lung-protective strategy to investigate whether R/I reflects the impact of PEEP on dynamic lung strain and lung recruitability assessed through the CT scan method.

## Methods

This study was conducted in the veterinary clinic of the University of Bari between June 2020 and September 2022, following approval of the Italian Ministry of Education, University and Research Committee (Prot. n.1234/2020-PR) on fourteen certified healthy mixed breeds of domestic pigs. All pigs were female and 6 months old and had a homogeneous weight of 47 [IQR 45–48] kg.

### Experimental protocol

At the beginning of the study, the animals were anesthetized, paralyzed, intubated, and mechanically ventilated (Servo-I, Getinge, Sweden). Anesthesia and muscle paralysis were obtained by continuous infusion of propofol (6 mg/kg/h) and cisatracurium (5 mcg/kg/min); analgesia was obtained by a single shot of buprenorphine (300 mcg). ARDS-like lung injury was produced by a 1-h intravenous infusion of a lipopolysaccharide membrane of *Escherichia coli* (LPS) (300 μg/kg) diluted in 20 ml of NaCl solution [23, 24]. Central venous and arterial lines were inserted through ultrasound guide. Continuous monitoring, including electrocardiography, heart rate, SpO_2_, end-tidal carbon dioxide and invasive arterial pressure, was kept for the whole study. Cardiac output (CO) was continuously monitored through the PRAM system (Vytech, USA).

Static lung CT scans were acquired during prolonged end-expiratory and end-inspiratory breath-holds (approximately 40 s) in the helical mode, thickness 5 mm, pitch = 1, rotation time 1.0 s, 120 KVP, 180 mAs, FOV 50 cm, and standard and chest convolution kernel (GE HiSPEED CT/e Dual, General Electric, New York, NY). The experimental protocol is summarized in the supplementary Fig. 1. Lung CT, respiratory mechanics, hemodynamics, and blood gas analysis results were obtained at the end of each experimental ventilation phases.

### Ventilation protocol and data acquisition

Constant flow volume-control ventilation was used for the whole study procedure. Respiratory rate (RR) was titrated to maintain pH within 7.35 and 7.45 and FiO_2_ was set to 1 for the whole study period. Three hours after beginning the LPS infusion, PEEP was set at 5 cmH_2_O (PEEP_LOW_ phase) for 1 h. Afterward, the PEEP was increased to reach a plateau end-inspiratory airway pressure (P_PLAT_) of 28–30 cmH_2_O according to the ExPress protocol [25] (PEEP_HIGH_ phase) while keeping V_T_ constant, for 1 h.

A Fleisch-type pneumotacograph (n.2, Metabo, Lausanne, Switzerland) and a pressure transducer (sample rate = 200 Hz) measured flow and airway pressure, respectively. V_T_ was calculated as the digital integration of the flow signal. The following parameters were recorded at the end of each PEEP step: V_T_, set PEEP, intrinsic PEEP (PEEP_i;st_) and P_PLAT_, the latter obtained through two second end-expiratory and end-inspiratory occlusions, respectively. Total PEEP (PEEP_TOT_) was computed as the sum of PEEP set and PEEP_i;st_. Driving pressure (ΔP) was calculated as (P_PLAT_ − PEEP_TOT_). Respiratory system compliance (C_RS_) was computed as the ratio between V_T_ and ΔP. The ventilatory ratio (Vr) was computed according to a standard formula described elsewhere [26]. Stress index (SI) was automatically calculated through the dedicated monitoring tool of the Servo-I Ventilator (Getinge, Sweden) [27]. All the signals were recorded and reviewed offline through dedicated software (ICU Lab, Kleistek, Bari, Italy).

### Recruitment-to-inflation ratio assessment

At the end of PEEP_HIGH_ phase, we performed a simplified, one-breath de-recruitment maneuver to estimate the difference in end-expiratory lung volume (EELV) between PEEP_HIGH_ and PEEP_LOW_ ($\Delta$ EELV) [20]. To do this, RR was set at 6 breaths/min while maintaining the inspiratory to expiratory time ratio unchanged (i:e = 1:2), thus obtaining an expiratory time of 6.6 s. Immediately after, PEEP_HIGH_ was abruptly decreased to PEEP_LOW_ and the exhaled volume during the prolonged expiration was recorded [20]. $\Delta$ EELV was calculated as the difference between the exhaled volume recorded during the one-breath de-recruitment maneuver and inspired tidal volume:

$$\Delta EELV=exhaled \,volume-{V}_T$$

Considering that $\Delta \text{EELV}$ includes two components: PEEP_HIGH_-induced recruited volume (V_REC_) and PEEP_HIGH_-induced inflation volume (*i.e*., the minimal predicted increase in lung volume due to PEEP_HIGH_) [21], the latter was calculated as the product of compliance at lower PEEP and the PEEP difference between the two steps:

$$PEEP_{HIGH - INFLATION \, VOLUME} = [C_{RS \, at \, PEEPlow} *(PEEP_{ HIGH} - PEEP_{ LOW} )]$$

Consequently, V_REC_ was computed as [19]:

$$V_{REC \, }= EELV_{{}} {-}PEEP_{HIGH - INFLATION \, VOLUME}$$

Finally, the compliance of recruited tissue (C_REC_) was obtained by:

$${C}_{REC}= \frac{{V}_{REC}}{{PEEP}_{HIGH}- {PEEP}_{LOW}}$$

and the recruitment-to-inflation ratio as the ratio between C_REC_ and C_RS_ at PEEP_LOW_ [19]:

$$R/I= \frac{{C}_{REC}}{{C}_{RS} at PEEPlow}$$

Of note, the R/I method mandates the assessment of any end-expiratory airway closure and of the corresponding airway opening pressure (AOP) [19]. To assess AOP, during ventilation at PEEP_LOW_ (5 cmH_2_O), RR was set at 5 breaths/min and the inspiratory time was adjusted to achieve flow rate of 5 L/minute while V_T_ was kept constant. During the procedure, that lasted a single breath, the time-airway opening pressure waveform was collected and analyzed on the ventilator screen. If present, AOP was identified as the inflection point on the pressure waveform above PEEP_LOW_ and used instead of PEEP_LOW_ for R/I calculation [19]. All the procedures for measuring R/I were verified trough a dedicated online tool (https://crec.coemv.ca*).*

### Computed tomography analysis

Quantitative CT scan analysis of all cranio-caudal CT scan slices above the diaphragm was performed using the Maluna software (Maluna version 2020, Goettingen, Germany) [17, 28]. For each slice, the entire left and right lungs were chosen as regions of interest by manually drawing their outer boundaries along the inside of the ribs and the inner boundaries along the mediastinal organs [17, 28]. The volume of each single voxel was computed as pixel area (0.35 mm^2^) times slice thickness (5 mm). The CT-Hounsfield unit (HU) number was used to define the density of each voxel [29, 30]. Given the voxel’s density and its relative volume, the voxel composition in gas and tissue (edema, interstitial water, blood, and lung structure) [29, 30] and the corresponding gas/tissue ratio (g/t) was automatically computed by the software [17, 28]. Thereby, the following Hounsfield unit (HU) ranges were used to define the different lung compartments: nonaerated, − 100 to + 100 HU; poorly aerated, − 101 to − 500 HU; normally aerated, − 501 to − 900 HU and hyperinflated, − 901 to − 1000 HU, while the volume of each compartment was obtained by multiplying the number of pixel found in each compartment to the voxel volume [17, 28, 29]. The total volume, tissue weight and gas volume of the entire lung are obtained by the Maluna software according to established methods [17, 28–30].

### Alveolar recruitment assessed through CT scan analysis

PEEP-induced tissue recruitment (T_REC_, grams) was measured as the difference in weight between PEEP_LOW_ and PEEP_HIGH_ of nonaerated lung tissue [29, 30] (online supplement; supplementary Fig. 2). To estimate the amount of recruited gas volume (GAS_REC_, milliliters), we replicated the method proposed by Chiumello et al. [31]. Briefly, the method assumes that the gas-to-tissue ratio (g/t) of the recruited tissue corresponds to the median g/t at PEEP_HIGH_ [31]. Accordingly, GAS_REC_ was computed as:

$$GAS_{REC} (ml) = T_{REC} (grams) \times \, g/t_{at \, PEEPHIGH}$$

Considering that: (1) the R/I normalizes C_REC_ to C_RS_ at PEEP_LOW_[19]_,_ and (2) compliance is an estimate of aerated lung size [32], we normalized T_REC_ and GAS_REC_ to total end-expiratory lung weight and end-expiratory total gas volume at PEEP_LOW_, respectively, as follow:

$$Normalized \, T_{REC} = T_{REC} /Total \, lung \, weight \, at \, PEEP_{LOW}$$

$$Normalized \, GAS_{REC} = GAS_{REC} /Total \, lung \, gas \, volume \, at \, PEEP_{LOW}$$

### Dynamic lung strain assessed through CT scan

In this study we considered PEEP_LOW_ (5 cmH_2_O) as the baseline condition, and therefore, the EELV at PEEP_LOW_ (EELV_LOW_) as FRC [15]. Based on this assumption, dynamic lung strain at PEEP_LOW_ was computed as the ratio between V_T_ and EELV_LOW_ [13, 15], while dynamic lung strain at PEEP_HIGH_ was computed as the ratio between V_T_ and EELV_LOW_ + GAS_REC_ [13, 15]:

$$Dynamic \,lung \,strain \,at \,{PEEP}_{LOW} =\frac{{V}_{T}}{{EELV}_{ LOW}}$$

$$Dynamic\, lung \,strain \,at \,{PEEP}_{HIGH} =\frac{{V}_{T}}{{(EELV}_{LOW}+ {GAS}_{REC})}$$

Static lung strain was computed in agreement with standard formula [13, 15]:

$$\text{Static lung strain}=\frac{{PEEP}_{VOLUME} }{({EELV}_{LOW} + {GAS}_{REC})}$$

where PEEP_VOLUME_ expresses the PEEP_HIGH_-induced inflation of already aerated lung regions at PEEP_LOW._

Having considered PEEP_LOW_ as baseline condition, we assumed that the static lung strain at PEEP_LOW_ was 0 [13, 15]. Then, we calculated PEEP_VOLUME_ at PEEP_HIGH_ as (DEELV − GAS_REC_) and the static lung strain as follows [13, 15]:

$$\text{Static lung strain at PEEP}_{\text{HIGH}}=\frac{\Delta EELV- {GAS}_{REC}}{{(EELV}_{LOW }+ {GAS}_{REC})}$$

Finally, global lung strain was calculated as the sum of static lung strain and dynamic lung strain [13, 15]*.*

### Endpoints

Primary endpoint: To determine whether the recruitment-to-inflation ratio reflects CT-scan measured PEEP-induced changes in dynamic strain.

Secondary endpoint: To determine whether R/I reflects CT-measured lung recruitability.

### Statistical analysis

For this experimental observational study, without performing a formal sample size calculation, we planned to enroll 14 animals as a convenient sample size, consistently with other studies on similar topics [27, 33, 34]. All numerical variables are expressed as medians and interquartile ranges [IQRs]. Shapiro–Wilk’s test was used to test normality and, based on its results, paired Student’s t-test or nonparametric paired Wilcoxon test were used to compare the two experimental conditions. The Mardia test was used to verify multivariate normality assumption, then correlations between continuous parameters were assessed with r-Pearson’s correlation coefficient (95% CI). For significant results, linear regression model was performed, and linearity of the relationship was verified by checking the normality of the residuals. Analysis of the potential outliers and potential highest influential points was checked by Cook's distance, Leverage-Residual plot and sensitivity analysis. A p value < 0.05 was considered to indicate statistical significance. Statistical analyses were performed using SAS/STAT® Statistics version 9.4 (SAS Institute, Cary, NC, USA).

## Results

### Respiratory mechanics, gas exchange and hemodynamics parameters

Table 1 reports respiratory mechanics, gas exchange and hemodynamic parameters in the two experimental conditions. PEEP_LOW_ was by protocol 5 cmH_2_O while median PEEP_HIGH_ resulting from the ExPress strategy was 20 cmH_2_O [18–21]. The median V_REC_ was 540 ml [368–762]. The median R/I was 1.08 [0.88–1.82]. At PEEP_HIGH_, compared to PEEP_LOW_, C_RS_ was 41 ml/cmH2O [32–50] versus 33 ml/cmH_2_O [23–42] (p = 0.037), stress index was 1.02 [1–1.03] versus 0.93 [0.82–0.97] (p = 0.001), PaO_2_/FiO_2_ was 167 mmHg [150–203] versus 108 mmHg [99–122] (p < 0.001), while PaCO_2_ and the ventilatory ratio remained unchanged. CO was 3.0 L/min [2.8–3.4] at PEEP_HIGH_ versus 3.9 L/min [3.5–4.2] at PEEP_LOW_ (p < 0.001).

**Table 1:** Respiratory mechanics, gas exchange and hemodynamics in the two different experimental PEEP-settings

	Low PEEP	High PEEP	p
Respiratory mechanics			
V_T_, ml/Kg _(PBW)_	6.8 [6.5–7]	6.8 [6.5–7]	NS
RR_,_ breaths/min	25 [22–30]	25 [22–30]	NS
PEEP_TOT_, cmH_2_O	5	20 [18–21]	< 0.0001
PEEP_i;st_, cmH_2_O	0	0	NS
P_PEAK_, cmH_2_O	25 [24–27]	35 [34–36]	< 0.0001
P_PLAT_, cmH_2_O	16 [15–20]	29 [28–30]	< 0.0001
C_RS_, ml/cmH_2_O	33 [23–42]	41 [32–50]	0.0379
Ventilatory Ratio	2.3 [2.4–2.9]	2.6 [2.2–3.1]	0.617
Stress Index	0.93 [0.82–0.97]	1.02 [1–1.03]	0.001
AOP	Not found	Not found	NS
PEEP-induced inflation volume	–	416 [328–569]	NS
V_REC_, ml	–	540 [368–762]	NS
C_REC,_ ml/cmH_2_O	–	40 [31–49]	NS
R/I	-–	1.08 [0.88–1.82]	NS
Gas exchange			
pH	7.36 [7.3–7.4]	7.36 [7.3–7.4]	NS
PaCO_2_, mmHg	57 [54–59]	61 [52–64]	0.691
FiO_2_, %	1	1	NS
PaO_2_/FiO_2_, mmHg	108 [99–122]	167 [150–203]	< 0.001
Hemodynamics			
Heart Rate, beats/min	100 [96–103]	114 [92–125]	0.113
MAP, mmHg	79 [68–92]	61 [56–70]	0.002
CO, L/min	3.9 [3.5–4.2]	3.0 [2.8–3.4]	< 0.001

Data are show as median [Inter Quartile Range]

V_T_: tidal volume; PBW: predicted body weight; RR: respiratory rate; PEEP_TOT_: total applied positive end-expiratory pressure; PEEPi_;st_: static intrinsic applied positive end-expiratory pressure; P_PEAK_: peak end-inspiratory airway pressure; P_PLAT_: plateau end-inspiratory airway pressure; C_RS_: compliance of respiratory system; AOP: Airways Opening Pressure; C_REC_: compliance of recruited volume; V_REC_: PEEP-induced recruited volume; C_REC_: compliance of recruited volume; R/I: recruitment to inflation ratio;PaCO_2_: arterial partial carbon dioxide pressure; PaO_2_: arterial partial oxygen pressure; FiO_2_: inspiratory oxygen fraction; MAP: mean arterial pressure; CO: cardiac output

### R/I and lung strain

**Fig. 1:**
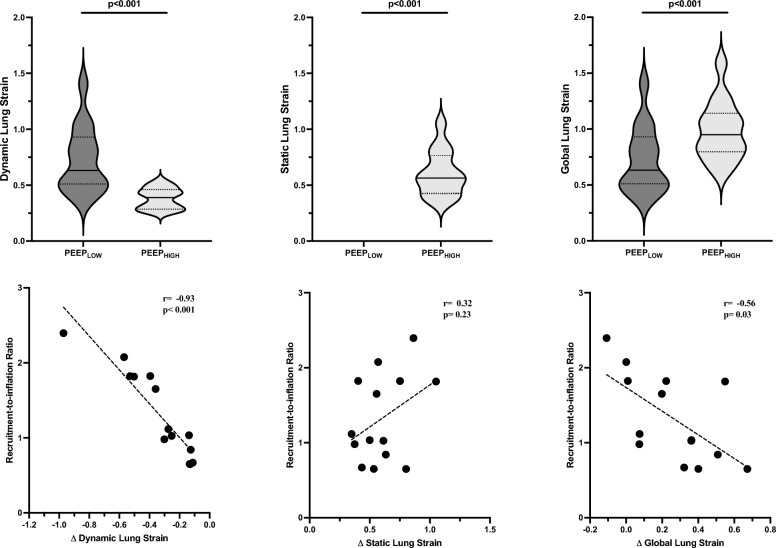
In the upper panel, box-violin graphs represent variation in global, dynamic and static lung strain going from PEEP_LOW_ (light gray) to PEEP_HIGH_ (dark gray). The lower panel depicts the correlations between recruitment-to-inflation (R/I) ratio and the changes in in global, dynamic and static lung strain going from PEEP_LOW_ to PEEP_HIGH_. The dotted line represents linear regressions, and each dot represents one pig

Figure 1 shows that going from PEEP_LOW_ to PEEP_HIGH_, dynamic lung strain decreased from 0.59 [0.56–0.80] to 0.37 [0.29–0.44] (p < 0.001), static lung strain increased from zero to 0.53 [0.44–0.75] (p < 0.001) and global lung strain increased from 0.59 [0.56–0.80] to 0.93 [0.76–1.42] (p < 0.001). The inter-individual coefficient of variation of dynamic lung strain reduction was 69%. R/I was strongly correlated with D dynamic lung strain (r = − 0.93; [95% CI − 0.78 to − 0.98] p < 0.001) and, less strongly, with D global lung strain (r = − 0.56; [95% CI − 0.05 to − 0.84], p = 0.03). No correlation was found between R/I and D static lung strain (r = 0.32; [95% CI −  0.23 to 0.74], p = 0.23). The regression model between R/I and D dynamic lung strain showed intercept and slope of respectively, + 0.52 (95% CI 0.29 to 0.77) and − 2.55 (95% CI − 3.19 to − 1.91).

### Lung recruitability assessed through the CT-scan method

**Fig. 2:**
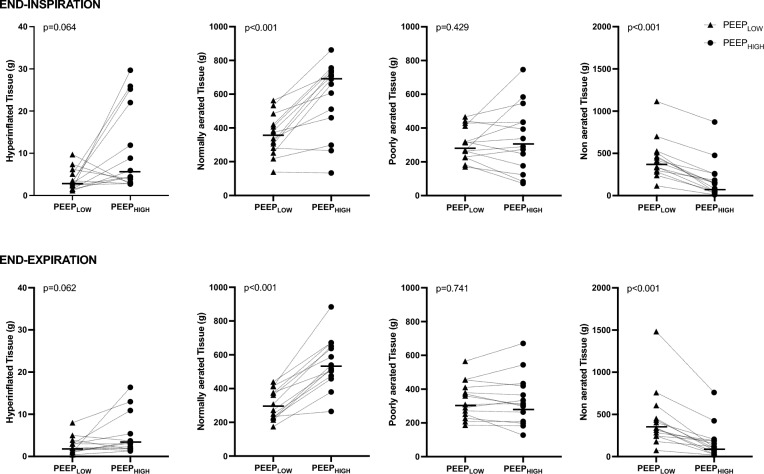
Individual values of hyperinflated, normally aerated, poorly aerated, and non-aerated lung tissue, going from PEEP_LOW_ to PEEP_HIGH_ at end-expiratory and end-inspiratory time

**Fig. 3:**
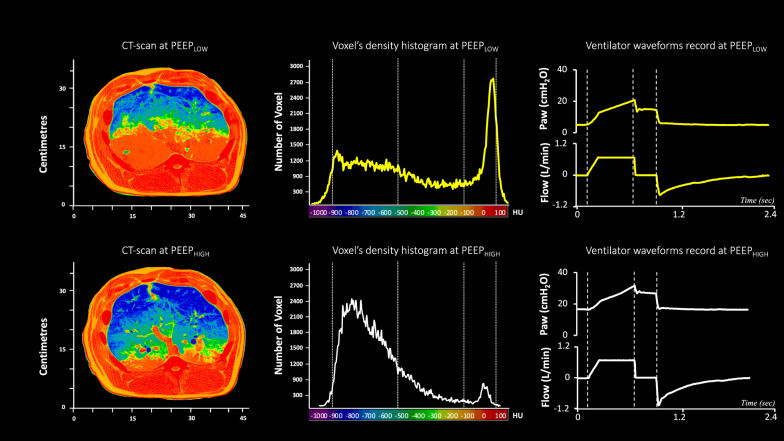
Left and middle panels: Representative computed tomography (CT) images and corresponding voxel density histograms of a large transverse lung section acquired under two different experimental ventilation conditions at end-expiration. Each image was interpreted using the UCLA color coding table (OsiriX image processing software, http://www.osirixfoundation.com, Geneva, Switzerland). Non-aerated lung tissue, ranging from − 100 to + 100 Hounsfield Units (HU), was depicted in shades of red (from dark red to orange), poorly aerated lung tissue (between − 500 to − 100 HU) was represented in shades of green, and normally aerated lung tissue (between − 900 to − 500 HU) was coded in dark and light blue. However, hyperinflated lung tissue (ranging from − 1000 to − 900 HU), which would have been represented in purple, was not observed upon raising PEEP from PEEP_LOW_ to PEEP_HIGH_. Right panel: experimental records in a representative animal showing the air flow and the opening airway pressure (Pao) traces during both experimental ventilation conditions. Dashed lines indicate the constant flow period

The absolute T_REC_ and GAS_REC_ were 246 g [182–288] and 385 ml [318–668], respectively, whereas the normalized T_REC_ and GAS_REC_ were 0.39 [0.25–0.55] and 0.85 [0.43–1.18], respectively. Table 2 and Fig. 2 report tissue weights and gas volumes of the different lung compartments (non-aerated, poorly aerated, normally aerated, hyperinflated) at end-expiration and end-inspiration, in the two experimental conditions. Figure 3 displays an experimental record of a representative animal, showing two lung CT slices acquired at end-expiration and the corresponding density histograms, in the two experimental conditions.

**Table 2:** Lung weight and gas volume of normally aerated, poorly aerated, nonaerated, and hyperinflated lung compartments at end-expiration and end-inspiration under each experimental ventilation condition

	Low PEEP	High PEEP	p
***End-expiration***			
*Lung weights*			
Total Lung Weight, g	979 [910–1175]	976 [915–1215]	0.726
Hyperinflated, g	2 [1–3]	3 [2–5]	0.062
Normally aerated, g	290 [231–376]	529 [475–651]	< 0.001
Poorly aerated, g	335 [257–402]	310 [209–419]	0.741
Nonaerated, g	408 [306–498]	146 [50–327]	< 0.001
*Gas-volumes*			
Total gas volume, ml	676 [441–793]	1735 [1382–1965]	< 0.001
Hyperinflated, ml	6 [4–10]	14 [12–21]	0.076
Normally aerated, ml	456 [306–618]	1424 [1099–1856]	< 0.001
Poorly aerated, ml	138 [96–176]	164 [119–303]	0.031
Nonaerated, ml	5 [2–9]	3 [1–6]	0.185
***End-inspiration***			
*Lung weights*			
Total Lung, g	988 [919–1231]	989 [923–1237]	0.753
Hyperinflated, g	3 [2–5]	5 [2–22]	0.062
Normally aerated, g	355 [281–420]	675 [460–731]	< 0.001
Poorly aerated, g	291 [228–412]	314 [176–435]	0.429
Nonaerated, g	388 [245–452]	114 [54–191]	< 0.001
*Gas-volumes*			
Total gas volume, ml	1748 [1325–2031]	2119 [1698–2481]	< 0.001
Hyperinflated, ml	11 [6–27]	46 [16–76]	0.014
Normally aerated, ml	880 [612–1073]	1786 [1358–2140]	< 0.001
Poorly aerated, ml	134 [85–184]	148 [84–331]	0.053
Nonaerated, ml	6 [3–8]	3 [1–11]	0.880

Data are show as median [Inter Quartile Range]

### R/I and lung recruitability

**Fig. 4:**
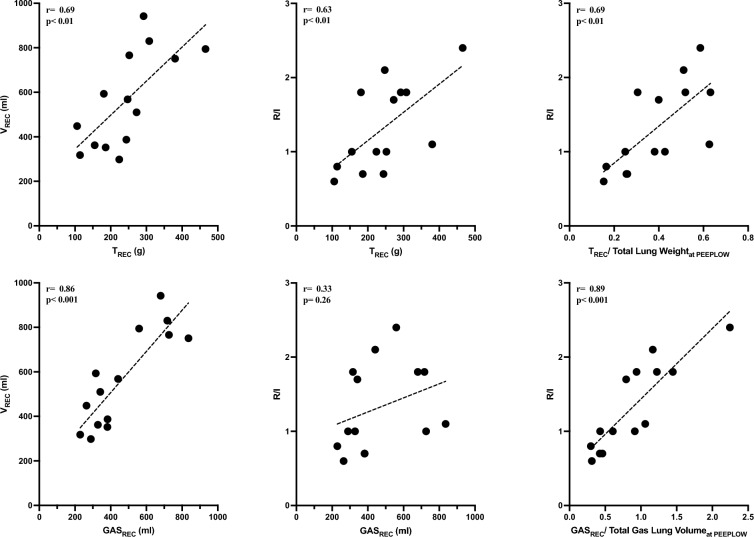
Left panels: Correlations between the absolute extent of PEEP-induced recruited volume (V_REC_) and tissue recruitment (T_REC_, upper panel) and gas recruitment (GAS_REC_, lower panel). Middle and right panels: Correlations between the recruitment-to-inflation ratio (R/I) and absolute and normalized values of T_REC_ (upper panel) and GAS_REC_ (lower panel). The dotted line represents linear regression, and each dot represents one pig

Figure 4 depicts the significant correlations between V_REC_ (measured with the R/I method) and GAS_REC_ and T_REC_ (measured with the CT scan method). Additionally, it shows that R/I was not correlated with absolute GAS_REC_ while was correlated with normalized GAS_REC_ (r = 0.89; [95% CI 0.71 to 0.97], p < 0.001), absolute T_REC_ (r = 0.63; [95% CI 0.14 to 0.87], p < 0.01) and normalized T_REC_ (r = 0.69; [95% CI 0.25 to 0.89], p < 0.01).

## Discussion

In this experimental study we found that R/I accurately reflects the impact of PEEP on dynamic lung strain and is closely correlated with normalized gas recruitment, as assessed through the “gold standard” CT scan method.

In patients with ARDS there is a consensus that higher PEEP levels should be applied only to “recruiters” [6] and accordingly, phenotyping patients according to the potential for lung recruitment would be advisable [35]. However, consensus on the clinical prediction of lung recruitability remains elusive. Various methods based on respiratory system compliance [36], oxygenation, shunts [37, 38] and pressure‒volume curves [21] have been proposed to determine the “best” PEEP. Large randomized control trials compared higher and lower PEEP settings setting PEEP according to physiological variables (oxygenation [37, 38], respiratory system compliance[25, 39], transpulmonary pressure [40]) but yielded disappointing results, showing no significant difference in terms of clinical benefit.

Recently, the R/I method has been introduced in clinical practice to predict lung recruitability through an easy-to-perform derecruitment maneuver, where the patient transitions from a higher to a lower PEEP level within a single breath, without being disconnected from the ventilator [20]. Unlike other respiratory mechanics-based methods, the R/I offers an estimate of recruited volume compared to the inflated volume. Based on the median R/I value in their original study patients cohort, Chen et al. identified R/I > 0.5 as a hallmark of “poor recruitment” and vice versa [19]. This suggests that when R/I is higher than 0.5, PEEP-induced inflation (increase in static strain) has a less injurious effect compared to the benefit of alveolar recruitment (decrease in dynamic lung strain), but further studies are needed to prove this assumption. However, it is important to consider that increasing PEEP, in addition to inducing a variable degree of alveolar recruitment, invariably generates static lung strain by distending previously aerated lung parenchyma (PEEP_VOLUME_). Another issue regards the wide PEEP transition to calculate R/I as originally proposed by Chen et al.; recently a more “granular” R/I measurement (i.e., calculating the R/I within narrower PEEP transitions) to identify the “best” compromise between recruitment and inflation [22].

From a physiological point of view, the impact of PEEP on dynamic lung strain depends on the ratio between the FRC at lower PEEP and PEEP-induced increase in FRC [13, 14]. We hypothesized that R/I may reflect the impact of PEEP on dynamic lung strain and documented a linear relationship between R/I and changes in dynamic lung strain (Fig. 1). However, hyperinflation may occur regardless recruitment. In this context, it is important to note that from a theorical viewpoint R/I cannot assess hyperinflation as it solely accounts for the ratio between the compliance of the recruited lung tissue (C_REC_) and total compliance at PEEP_LOW_ (C_RS_) [19]. To substantiate this concept, we have performed a supplementary analysis showing that individual R/I and PEEP-induced hyperinflation are not correlated (r = 0.04; p = 0.89) (online supplement; Supplementary Fig. 3). Furthermore, the R/I is unsuitable to assess regional tidal hyperinflation. Accordingly, when setting high PEEP, hyperinflation (both global and regional) should be assessed regardless of the R/I value to achieve a fully protective ventilatory strategy.

The ability of R/I to predict PEEP-induced alveolar recruitment has been validated since now with respiratory mechanics [19]. However, CT-scan remains the gold standard for measuring PEEP-induced alveolar recruitment by quantifying T_REC_ [12] and GAS_REC_ [31]. In our study, we observed poor correlations between R/I and absolute T_REC_ and no correlation between R/I and GAS_REC_ (Fig. 4). Nevertheless, upon reflection, we reasoned that (a) R/I normalizes the compliance of PEEP-recruited lung tissue (C_REC_) to the total compliance at PEEP_LOW_ (C_RS_); (b) compliance is an estimation of aerated lung size [32] and, finally, (c) recent studies have found that at low PEEP/ZEEP C_RS_ is an estimate of FRC [41] and indeed we found a good correlation between C_RS_ and total end-expiratory gas volume at PEEP_LOW_ (r = 0.75; p < 0.01) (supplementary Fig. 7). Thus, we hypothesized that the correct correlation should have been between R/I and GAS_REC_ normalized to total lung capacity at PEEP_LOW_. When we conducted this correlation, we found a significant and strong relationship (r = 0.89, p < 0.001), as depicted in Fig. 4. We extended this reasoning to T_REC_ normalized to total lung weight at PEEP_LOW_ (r = 0.69, p < 0.01, Fig. 4). Additionally, we explored the relationship between V_REC_ and T_REC_ and GAS_REC_ measured with CT scan and, again, found significant correlations (r = 0.69 and r = 0.86 respectively, both p < 0.01, see Fig. 4). Overall, these findings seem the first to validate the performance of R/I against the gold standard CT-scan technique.

From a methodological point of view, it is important to discuss that the method to quantify PEEP-induced alveolar recruitment through CT scan is not uniformly agreed upon [42]. In our study, we quantified recruitment according to Gattinoni et al. [12] as PEEP-induced re-aeration of non-aerated lung tissue (weight of recruited tissue = weight of non-aerated tissue at PEEP_LOW_ – weight of non-aerated tissue at PEEP_HIGH_). However, another approach considers PEEP-induced differences in non-aerated plus poorly aerated lung tissues [49]. Nevertheless, when we applied both these approaches to quantify T_REC_, we found no significant difference and a good correlation between the two methods (r = 0.82; p < 0.01). Also, the quantification of GAS_REC_, lung strain, and the correlations between the R/I and these variables were not affected by the method to calculate T_REC_. We present these results in the online supplement (see online supplement Figs. 4, 5 and 6).

In our study, we used the approach employed in the ExPress trial to set PEEP_HIGH_ (increasing PEEP up to a plateau pressure of 30 cmH_2_O) [25]. However, Chiumello and colleagues demonstrated that the ExPress PEEP-setting strategy does not consistently correlate with lung recruitability and is associated with higher risks of hyperinflation in patients with the higher quartile of hyperinflated tissue on CT scans [43]. Nevertheless, we point out that increasing PEEP to enhance lung recruitability is a well-established practice in the management of moderate-to-severe acute respiratory distress syndrome [6] and, furthermore, recently Protti et al. showed in COVID-related ARDS (CARDS) that when higher PEEP levels are applied to patients with high recruitability (similar to our experimental model), the balance between recruitment and hyperinflation is towards recruitment [44]. Finally, an ongoing multicenter trial designed to individualize PEEP based on the R/I approach adopts a PEEP-setting strategy similar to that of the ExPress trial [45].

Our study has limitations. First, it was conducted in a highly recruitable model of ARDS, which limits the generalizability of our findings. However, if confirmed by clinical studies, our experimental data may prove useful in supporting PEEP settings in the significant subset of highly recruitable patients. Second, we did not conduct an a priori sample size calculation for this study. However, from a post hoc power analysis resulted that for a Type III F test of one predictor in a regression model with a significance level of 0.05, a sample size of 14 has a power > 0.99 to detect a R-square of 0.87 between the tested predictor (Delta Dynamic Lung Strain) and response (R/I). Third, our results were obtained in the supine position, which does not necessarily imply their reproducibility in the prone position [46]. Fourth: Our results may have been influenced by the different approaches between CT scan and R/I assessments. Indeed (see Methods and Supplemental Fig. 1), by experimental design, we performed a CT scan at low PEEP followed by a CT scan at high PEEP, whereas the R/I was obtained by suddenly decreasing PEEP from a high to a low level, as per the R/I protocol. These different approaches could have influenced the "volume history" of our model and certainly may represent a study bias, even though the CT scans were taken after 1 h of application of each PEEP level, suggesting a stable condition. Probably, a brief recruiting maneuver applied when moving from the lower to the higher PEEP level could have helped to resolve this point. Fifth: manually delineating CT regions of interest may have introduced a potential bias in the CT-scan analysis. However, this approach aligns with similar investigations in animal models [17]. Finally, we do not provide data about blood or lung samples assessing inflammation and VILI in the two experimental conditions, so we can only speculate on the eventual impact of higher PEEP on VILI.

## Conclusions

In a highly recruitable model of ARDS, we found that the R/I reflects the impact of PEEP on dynamic lung strain and normalized gas recruitment, as assessed through the CT-scan method.

### Supplementary Information

Supplementary Material 1.

## Data Availability

The study dataset used is available upon a justified request.

## Abbreviations

PEEP: Positive end-expiratory pressure
ARDS: Acute respiratory distress syndrome
VILI: Ventilator-induced lung injury
CT: Computed tomography
R/I: Recruitment to-inflation ratio
PLR: Potential for lung tissue recruitment
FRC: Functional residual capacity
V_T_: Tidal volume
P_PLAT_: Plateau end-inspiratory airway pressure
ΔP: Driving pressure
C_RS_: Compliance of respiratory system
SI: Stress index
CO: Cardiac output
LPS: Lipopolysaccharide
RR: Respiratory rate
C_REC_: Compliance of tissue recruited
V_REC_: Recruited volume
EELV: End-expiratory lung volume
AOP: Airway opening pressure
