# Supplementary material for: Recruitment-to-inflation ratio reflects the impact of peep on dynamic lung strain in a highly recruitable model of ARDS
Source: Ann Intensive Care. 2024 Jul 4;14:106. doi: 10.1186/s13613-024-01343-w (PMC11224186; doi:10.1186/s13613-024-01343-w)

ONLINE

SUPPLEMENT

RECRUITMENT-TO-INFLATION RATIO REFLECTS THE IMPACT OF PEEP ON DYNAMIC LUNG STRAIN IN A HIGHLY RECRUITABLE MODEL OF ARDS

Francesco Murgolo[1]* M.D., Domenico L. Grieco[2,3]* M.D., Savino Spadaro[4] Ph.D., Nicola Bartolomeo[5] Ph.D., Rossella di Mussi[1] Ph.D., Luigi Pisani[1] Ph.D., Marco Fiorentino[6] M.D., Alberto Maria Crovace[7] Ph.D, Luca Lacitignola[8] Ph.D., Francesco Staffieri[8] Ph.D., Salvatore Grasso[1] M.D.

* These authors are first co-authors.

1. Department of Precision-Regenerative Medicine and Jonic Area (DiMePRe-J), Section of Anesthesiology and Intensive Care Medicine, University of Bari "Aldo Moro", Bari, Italy.
2. Department of Anesthesia, Intensive Care and Emergency. Fondazione Policlinico A. Gemelli IRCCS. Rome, Italy
3. Department of Anesthesiology and Intensive Care Medicine, Catholic University of The Sacred Heart. Rome, Italy
4. Department of Translational Medicine, Section of Anesthesiology and Intensive Care Medicine, University of Ferrara, Italy.
5. Interdisciplinary Department of Medicine, University of Bari "Aldo Moro", Bari, Italy.
6. Nephrology, Dialysis and Transplantation Unit, Department of Precision and Regenerative Medicine and Ionian Area (DiMePRe-J), University of Bari, Bari, Italy
7. Department of veterinary medicine, University of Sassari, Sassari, Italy
8. Department of Precision-Regenerative Medicine and Jonic Area (DiMePRe-J), Section of Veterinary Medicine, University of Bari "Aldo Moro", Bari, Italy.

TABLE OF CONTENTS

1. **ADDITIONAL METHOD**……………………………………………..………...…..…………..3
2. **ADDITIONAL RESULTS**……………………………………………..………...…..…………..5
3. **REFERENCES**………………………………………………….....………...…..…………..6
4. **TABLE 1-OS**…………………………………………….………...…..………………......8
5. **SUPPLEMENTARY FIGURES** …………………………………………….……….....9

ADDITIONAL METHOD

Alveolar recruitment and lung strain assessed through CT scan analysis.

To assess the extent of PEEP-induced lung recruitment through CT-scan, we employed two different methods to estimate the weight of recruited lung tissue (T_{REC}). The first approach (method A) considers T_{REC} as the absolute decrease in non-aerated lung tissue weight upon raising PEEP $PEEP_{LOW}$ to $PEEP_{HIGH}$ (i.e. $T_{REC(NON)}$) [1] . The second approach (method B) takes into account the non-aerated *plus* poorly-aerated tissue weight [2] (*i.e.* $T_{REC(NON + POORLY)}$). Since the computation of T_{REC} is crucial for deriving the amount of gas entering newly recruited tissue (GAS_{REC}) and consequently static lung strain at $PEEP_{HIGH}$, we adjusted all the formulas as follow:

Tissue recruitment

PEEP-induced tissue recruitment (T_{REC}, grams) was measured as:

Method A (taking into account non-aerated tissue only):

- $T_{REC}\,(non);\, grams = NA_{at\ PEEPLOW} -= NA_{at\ PEEPHIGH}$

Method B (taking into account non-aerated + poorly aerated tissue):

- $T_{REC}\,(non{+}poorly);\, grams = (NA_{at\ PEEPLOW} + PA_{at\ PEEPLOW}) - (NA_{at\ PEEPHIGH} + PA_{at\ PEEPHIGH})$

Where $NA_{at\ PEEPLOW}$ *and* $PA_{at\ PEEPLOW}$ represent respectively the weight of non-aerated and poorly aerated tissue at $PEEP_{LOW}$ and $NA_{at\ PEEPHIGH}$ *, and* $PA_{at\ PEEPHIGH}$ the weight of non-aerated and poorly aerated tissue at $PEEP_{HIGH}$.

The two T_{REC}s were normalized to total end-expiratory lung weight as follow:

$$Normalized\ T_{REC(NON)} = T_{REC(NON)}\ /\ Total\ lung\ weight\ at\ PEEP_{LOW}$$

$$Normalized\ T_{REC(NON+POORLY)} = T_{REC(NON+POORLY)}\ /\ Total\ lung\ weight\ at\ PEEP_{LOW}$$

Gas recruitment

PEEP-induced gas recruitment (GAS_{REC}, ml) was measured as follows:

Method A : $GAS_{REC}\ (non);\ ml = T_{REC}\ (non)\ x\ g/t_{at\ PEEPHIGH}$

Method B : $GAS_{REC}\ (non{+}poorly);\ ml = T_{REC}\ (non{+}poorly)\ x\ g/t_{at\ PEEPHIGH}$

Where $g/t_{at\ PEEPHIGH}$ is the median gas-to-tissue ratio at $PEEP_{HIGH}$

The two GAS_{REC}s were normalized to total end-expiratory lung volume as follows:

$$Normalized\ GAS_{REC(NON)} = T_{REC(NON)}\ /\ Total\ lung\ volume\ at\ PEEP_{LOW}$$

$$Normalized\ GAS_{REC(NON+POORLY)} = T_{REC(NON+POORLY)}\ /\ Total\ lung\ volume\ at\ PEEP_{LOW}$$

Lung strain

At $PEEP_{HIGH}$, static and dynamic lung strain were measure as :

Method A:

- $Dynamic\ lung\ strain\ at\ PEEP_{HIGH} = \frac{V_T}{(EELV_{LOW} + GAS_{REC}(non)}$
- $Static\ lung\ strain\ at\ PEEP_{HIGH} = \frac{PEEP_{VOLUME}}{(EELV_{LOW} + GAS_{REC}(non)}$

Method B:

- $Dynamic\ lung\ strain\ at\ PEEP_{HIGH} = \frac{V_T}{(EELV_{LOW} + GAS_{REC}(non{+}poorly)}$
- $Static\ lung\ strain\ at\ PEEP_{HIGH} = \frac{PEEP_{VOLUME}}{(EELV_{LOW} + GAS_{REC}(non{+}poorly)}$

Global lung strain was calculated as the sum of static lung strain and dynamic lung strain.

ADDITIONAL RESULTS

Correlation between individual R/I and PEEP-induced hyperinflation

Supplementary Figure 3 shows the variation in the weight of hyperinflated lung tissue passing from $PEEP_{LOW}$ to $PEEP_{HIGH}$ during both the end-expiration and end-inspiration phases in each animal. No correlation was found between R/I and change in hyperinflated lung tissue weight at end-expiratory and end-inspiratory times (r=0.04, p=0.89 and r=0.11, p=0.68; respectively).

Comparisons between Different Methods in Assessing Recruitment through CT scanning

Table 1-OS reports T_{REC}, GAS_{REC} and dynamic, static and global lung strain at $PEEP_{HIGH}$ derived from the two different approaches to the calculation of lung recruitment (see supplemental methods). We found minimal and non-significant differences between the absolute values of T_{REC}s and GAS_{REC}s calculated with the two methods (246g [182-288] *vs* 241g [196-310], p=0.74 and 385 ml [318-668] *vs* 460 ml [343-699], p=0.59), as well as between their normalized values. The quantifications of global, static and dynamic lung strains were not substantially affected by the method to calculate GAS_{REC}. **Supplementary figure 4** shows the correlation between $T_{REC(NON)}$ and $T_{REC(NON+POORLY)}$ (r=0.82;p<0.001) and between $GAS_{REC(NON)}$ and $GAS_{REC(NON+POORLY)}$ (r=0.83;p<0.001). **Supplementary figure 5** and **Supplementary figure 6** replicate **figure 1** and **figure 4** of the main manuscript, respectively, indicating the results obtained through the two calculation methods of TREC and GAS_{REC}.

Correlations between respiratory system compliance and total lung weight and gas volume

Since R/I normalizes the compliance of PEEP-recruited lung tissue (C_{REC}) to the total compliance at $PEEP_{LOW}$ (C_{RS}) and since C_{RS} is an estimation of aerated lung size [3] and, finally, since it has been recently showed that at low PEEP/ZEEP C_{RS} is indeed an estimate of FRC [4], we

investigated the relationship between C_{RS} at $PEEP_{LOW}$ and total lung weight (grams) and gas volume (milliliters) at $PEEP_{LOW}$ measured at end-expiration. Confirming the concept that C_{RS} is an estimation of aerated lung size, particularly at ZEEP/LOW PEEP we found a pretty good and significant correlation between C_{RS} and total end-expiratory gas volume at $PEEP_{LOW}$ ($r=0.75; p<0.01$) but no correlation between C_{RS} and total end-expiratory lung weight at $PEEP_{LOW}$ ($r=-0.15; p=0.59$) (s**upplementary figure 7).**

ADDITIONAL REFERENCES

1. Gattinoni L, Caironi P, Cressoni M, Chiumello D, Ranieri VM, Quintel M, et al. Lung recruitment in patients with the acute respiratory distress syndrome. N Engl J Med. 2006;354:1775–86.

2. Malbouisson LM, Muller JC, Constantin JM, Lu Q, Puybasset L, Rouby JJ, et al. Computed tomography assessment of positive end-expiratory pressure-induced alveolar recruitment in patients with acute respiratory distress syndrome. Am J Respir Crit Care Med [Internet]. Am J Respir Crit Care Med; 2001 [cited 2023 Oct 13];163:1444–50. Available from: https://pubmed.ncbi.nlm.nih.gov/11371416/

3. Gattinoni L, Marini JJ, Pesenti A, Quintel M, Mancebo J, Brochard L. The "'baby lung'" became an adult. Intensive Care Med. 42.

4. Grassi A, Teggia-Droghi M, Borgo A, Szudrinsky K, Bellani G. Feasibility of Setting the Tidal Volume Based on End-Expiratory Lung Volume: A Pilot Clinical Study. Crit Care Explor [Internet]. Wolters Kluwer Health; 2024 [cited 2024 May 14];6:e1031. Available from: /pmc/articles/PMC10793974/

TABLE 1-OS

Comparisons between Different Methods in Assessing tissue recruitment, gas recruitment and different lung strains at $PEEP_{HIGH}$.

	Method A	Method B	p
T_{REC}	246 [182-288]	241 [196-310]	0.74
GAS_{REC}	385 ml [318-668]	460 [343-699]	0.59
Normalized T_{REC}	0.39 [0.25-0.55]	0.43 [0.26-0.56]	0.27
Normalized GAS_{REC}	0.85 [0.43-1.18]	0.91 [0.52-1.22]	0.39
Dynamic lung strain at $PEEP_{HIGH}$	0.37 [0.29-0.44]	0.35 [0.29 - 0.46]	0.62
Static lung strain at $PEEP_{HIGH}$	0.53 [0.44 -0.75]	0.53 [0.43 -0.75]	0.62
Global Lung strain at $PEEP_{HIGH}$	0.93 [0.74-1.42]	0.92 [0.72-1.45]	0.61

SUPPLEMENTARY FIGURES

Supplementary figure 1

Flow chart of the experimental ventilation protocol. Constant flow volume-control ventilation was used for the whole study procedure. During the 2-hour induction of ARDS-like lung damage, positive end-expiratory pressure (PEEP) was set to 0 cmH_2O, tidal volume (V_T) was maintained at 6-8 millilitres per kilogram of predicted body weight (PBW), respiratory rate (RR) was titrated to maintain pH between 7.35 and 7.45 and FiO_2 was set to 1. Three hours after the start of mechanical ventilation, a PEEP trial was conducted: V_T, RR and FiO_2 were kept constant, PEEP was initially set at 5 cmH_2O ($PEEP_{LOW}$ phase) for one hour and then increased to achieve a plateau pressure of 28-30 cmH_2O ($PEEP_{HIGH}$ phase) for an additional hour. End-expiratory CT scans, lung mechanics, hemodynamics and arterial blood gas analysis were obtained at the end of each PEEP step. After the $PEEP_{EXPRESS}$ phase, a single-breath derecruitment maneuver was performed by decreasing $PEEP_{HIGH}$ to $PEEP_{LOW}$ to assess the recruitment to inflation ratio (R/I).

Supplementary figure 2

Representative computed tomography (CT) images acquired at a level resulting in the largest transverse lung section between the most cranial point of the diaphragm and the base of the heart under different PEEP-experimental conditions at end-expiration. Red dashed line delineates the the non-aerated tissue at $PEEP_{LOW.}$ Yellow dashed line and area represent the transposition of the recruited tissue at $PEEP_{HIGH}$.

Supplementary figure 3

Correlation between individual R/I and PEEP-induced hyperinflation. In the upper panels, individual values of hyperinflated lung tissue, going from $PEEP_{LOW}$ to $PEEP_{HIGH}$ at end-expiratory and end-inspiratory time. In the lower panels, relationship between recruitment to inflation ratio (R/I) and degree of variation (delta) of hyperinflated lung tissue passing from $PEEP_{LOW}$ to $PEEP_{HIGH.}$ Each colored dot represents one pig.

Supplementary figure 4

In the upper panel, box-violin graphs represent variation in global, dynamic and static lung strain going from $PEEP_{LOW}$ to $PEEP_{HIGH}$. *Dark gray boxes* identify various lung strains computed by considering gas recruitment as the gas entering newly recruited lung tissue (absolute decrease of non-aerated tissue weight between $PEEP_{LOW}$ to $PEEP_{HIGH}$), *light gray boxes* identify various lung strains computed by considering gas recruitment as the gas entering newly recruited tissue (absolute decrease in the sum of non-aerated *plus* poorly aerated tissue weight between $PEEP_{LOW}$ to $PEEP_{HIGH}$). The lower panel depicts the correlations between recruitment-to-inflation (R/I) ratio and the changes in in global, dynamic and static lung strain going from $PEEP_{LOW}$ to $PEEP_{HIGH}$. The dotted line represents linear regressions, and each dot represents one pig.

Supplementary figure 5

In the right panels: Correlations between absolute PEEP-induced recruited volume (V_{REC}) and absolute tissue recruitment (T_{REC}) (*upper panel*) and absolute gas recruitment (GAS_{REC}) (*lower panel*). In the might panels: Correlations between recruitment-to-inflation ratio (R/I) and absolute T_{REC} (*upper panel*) and absolute GAS_{REC} (*lower panel*). In the right panels: Correlations between R/I and normalized T_{REC} (*upper panel*) and normalized GAS_{REC} (*lower panel).* PEEP-induced recruited volume was obtained through a simplified de-recruitment maneuver by abruptly lowering PEEP from

$PEEP_{HIGH}$ to $PEEP_{LOW}$ within a single breath. Tissue and gas recruitment were assessed by the CT-scan method: $T_{REC(NON)}$ was computed as the absolute decrease of non-aerated lung tissue weight between $PEEP_{LOW}$ to $PEEP_{HIGH.}$ $T_{REC(NON+POORLY)}$ was computed as the absolute decrease in the sum of non-aerated *plus* poorly aerated tissue weight between $PEEP_{LOW}$ to $PEEP_{HIGH.}$ $GAS_{REC(NON)}$ and $GAS_{REC(NON+POORLY)}$ were obtained by multiplying $T_{REC(NON)}$ and $T_{REC(NON+POORLY)}$ to gas/tissue ratio at $PEEP_{HIGH,}$ respectively. Normalized T_{REC}s and normalized GAS_{REC}s were obtained by dividing the absolute values of T_{REC} and GAS_{REC} to the total end-expiratory lung tissue weight and gas volume at $PEEP_{LOW,}$ respectively. The dotted line represents linear regression, and each dot represents one pig.

Supplementary figure 6

Correlation between different method to assess tissue recruitment (left panel) and gas recruitment (right panel) through CT scanning. $T_{REC(NON)}$ was computed as the absolute decrease of non-aerated lung tissue weight between $PEEP_{LOW}$ to $PEEP_{HIGH.}$ $T_{REC(NON+POORLY)}$ was computed as the absolute decrease in the sum of non-aerated *plus* poorly aerated tissue weight between $PEEP_{LOW}$ to $PEEP_{HIGH.}$ $GAS_{REC(NON)}$ and $GAS_{REC(NON+POORLY)}$ were obtained by multiplying $T_{REC(NON)}$ and $T_{REC(NON+POORLY)}$ to gas/tissue ratio at $PEEP_{HIGH,}$ respectively.

Supplementary figure 7

Correlation between respiratory system compliance at $PEEP_{LOW}$ and total tissue lung weight at $PEEP_{LOW}$ (*left panel*) and total gas volume at $PEEP_{LOW}$ (*right panel*). The dotted line represents linear regression, and each dot represents one pig.

Supplementary figure 8

Individual values of hyperinflated, normally aerated, poorly aerated, and non-aerated lung tissue, going from $PEEP_{LOW}$ to $PEEP_{HIGH}$ at end-expiratory and end-inspiratory time. Each colored dot represents one pig.

Supplementary Figure 1

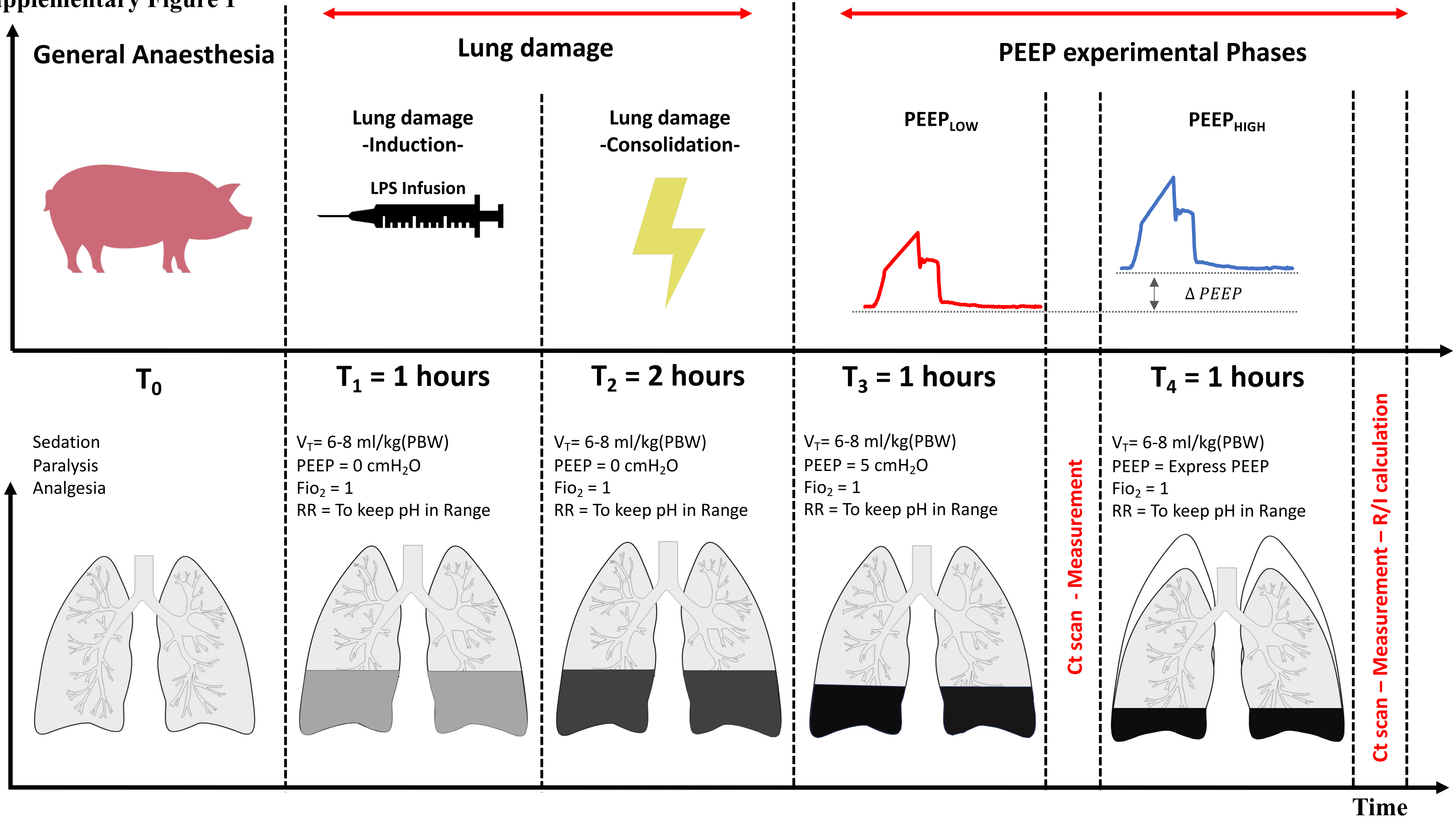

Supplementary figure 2

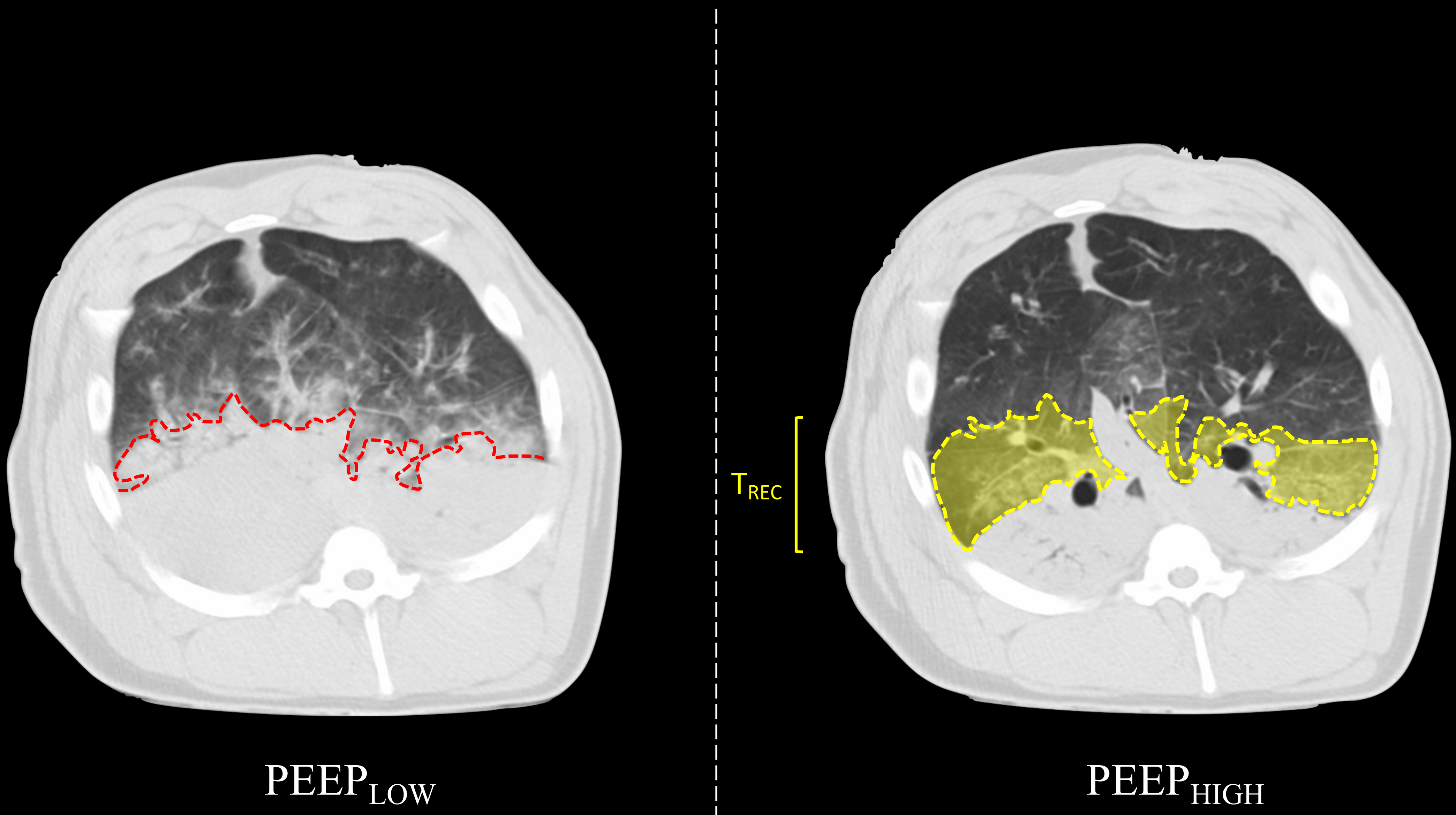

Supplementary figure 3

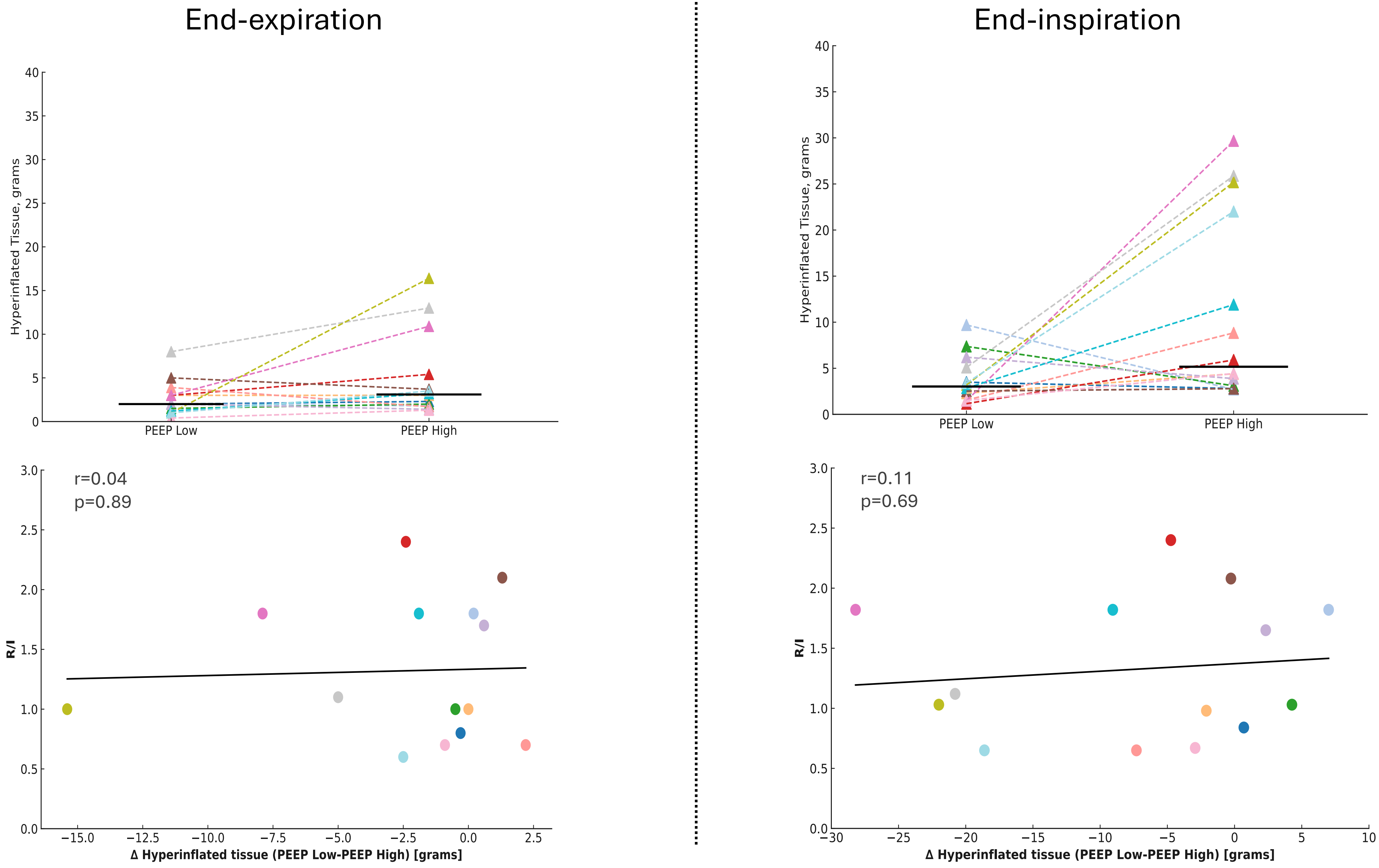

Supplementary figure 4

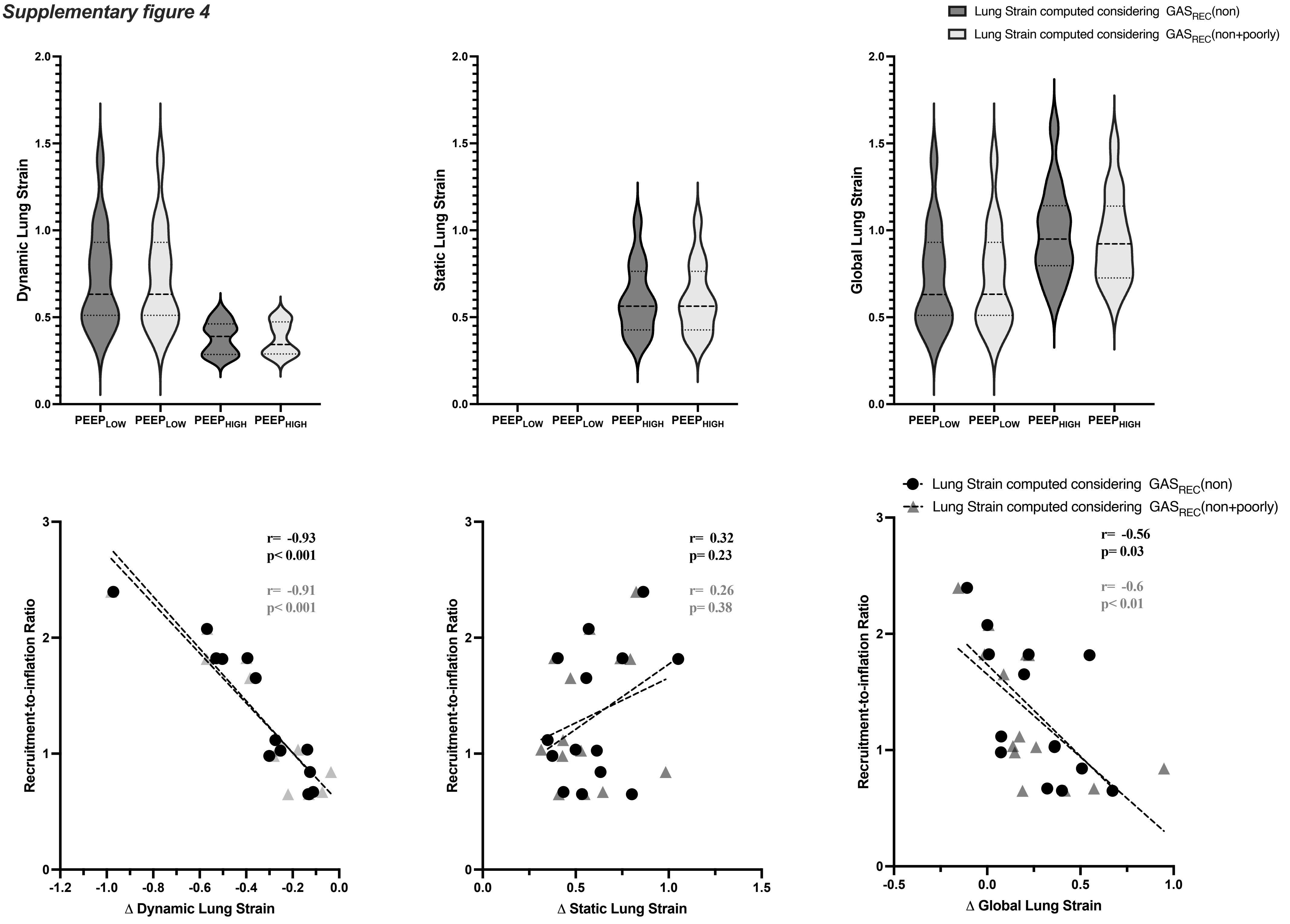

Supplementary figure 5

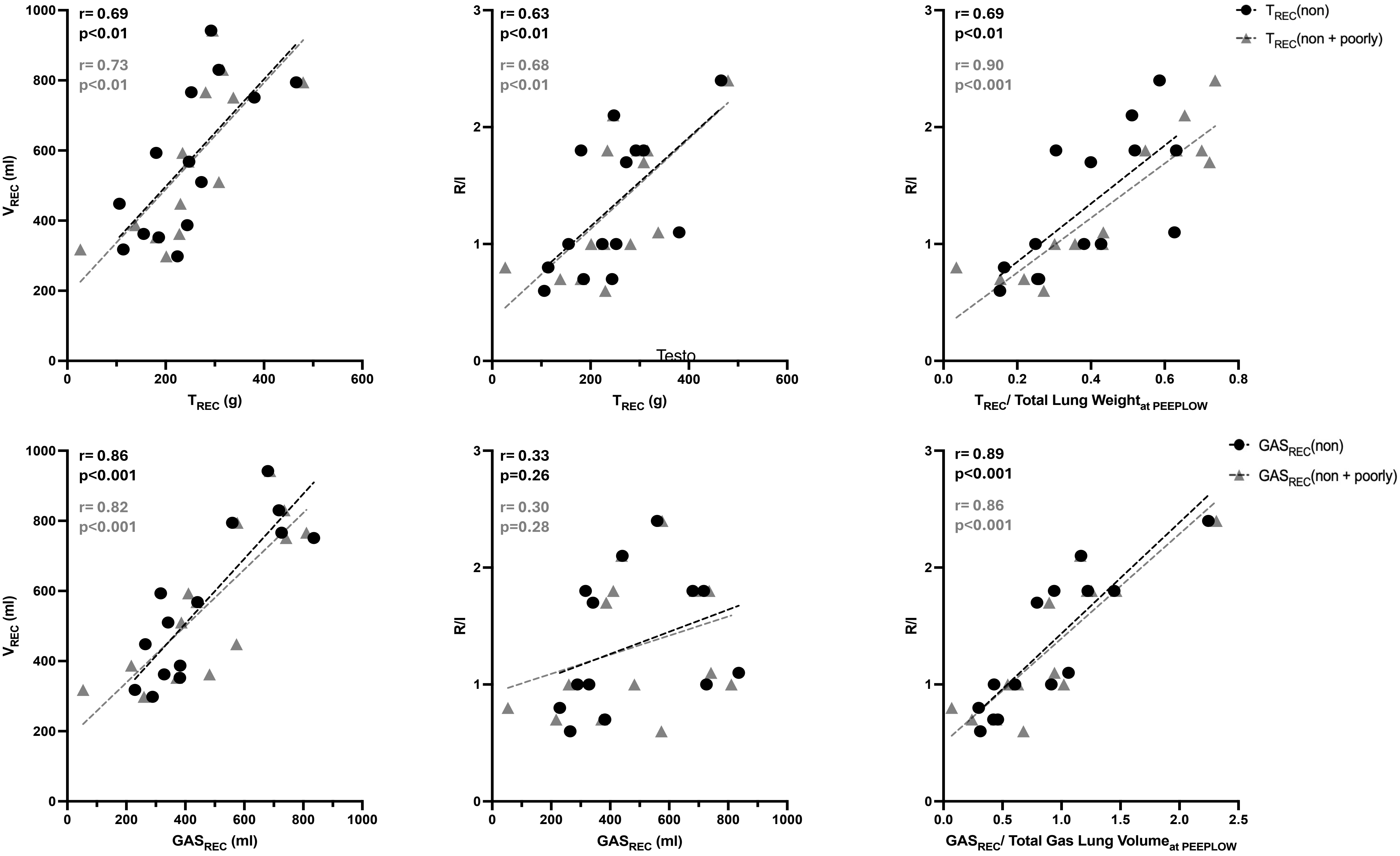

Supplementary figure 6

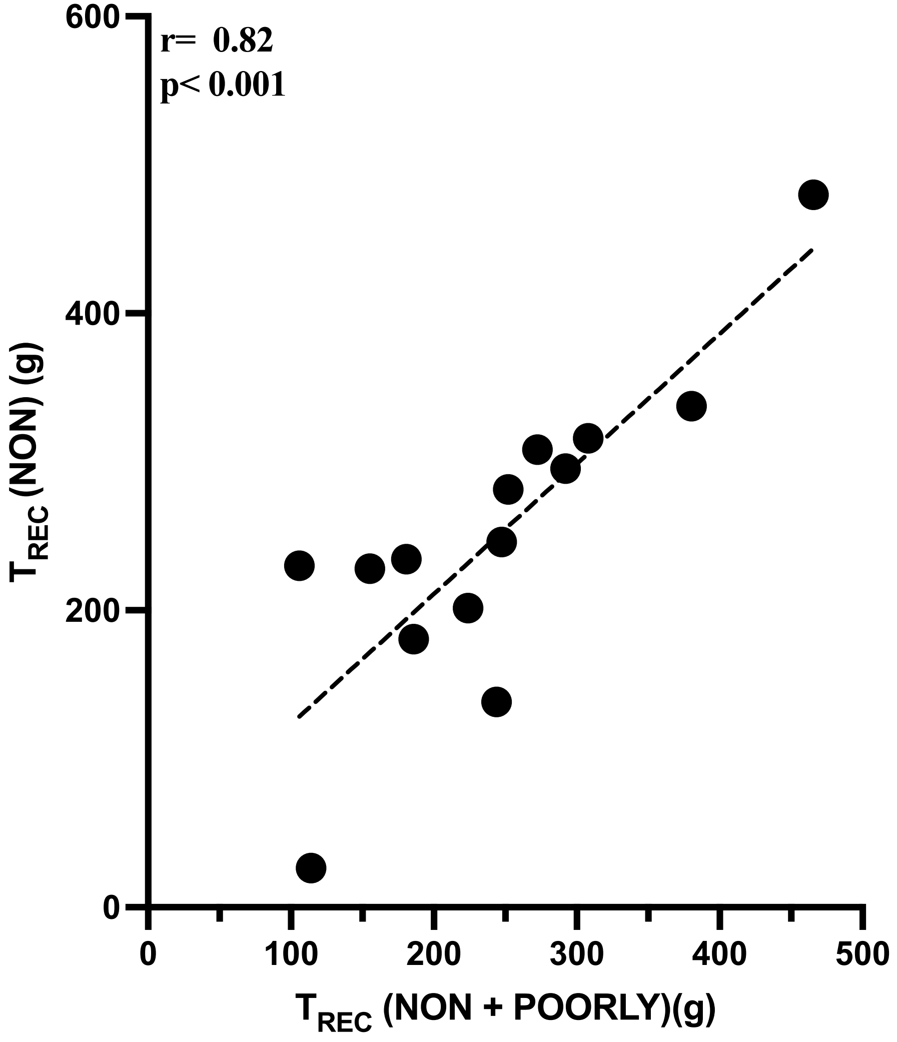

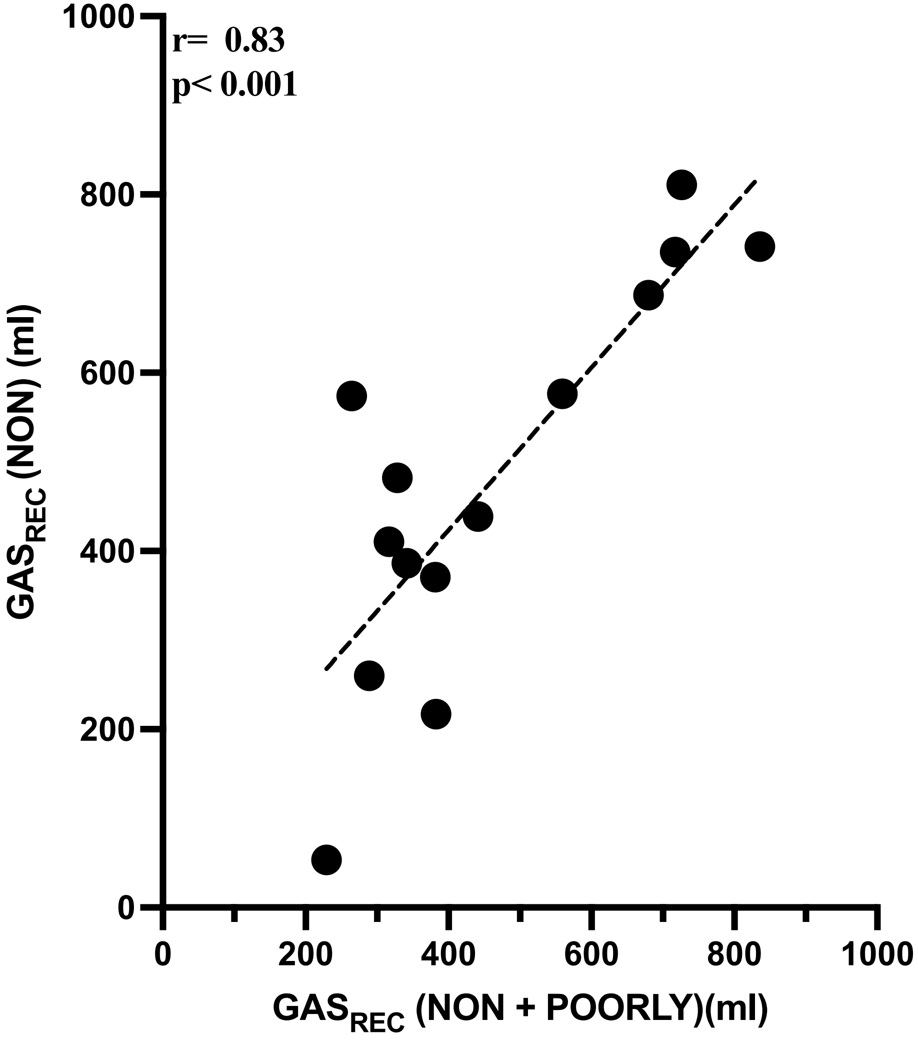

Supplementary figure 7

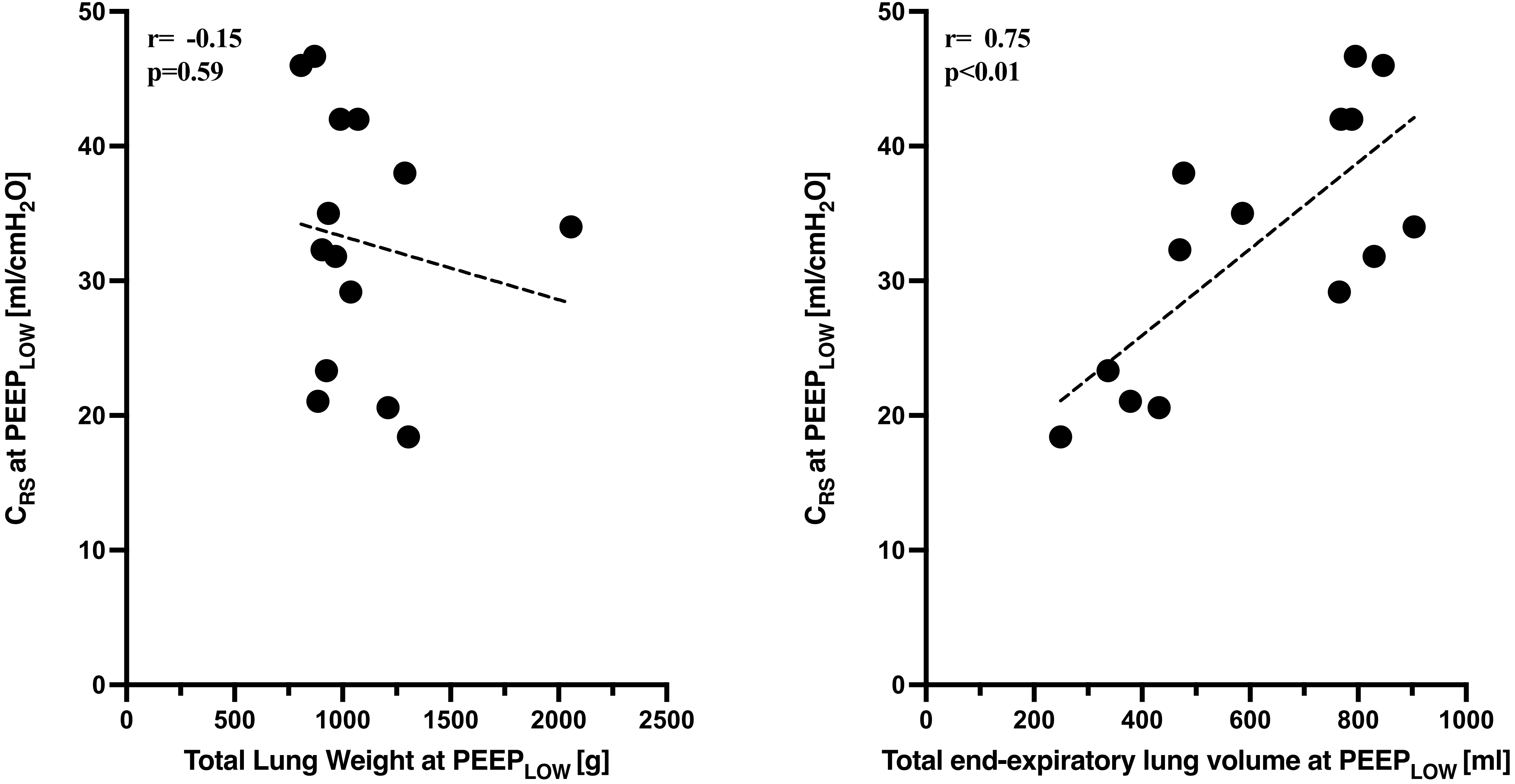

Supplementary figure 8

END-INSPIRATION

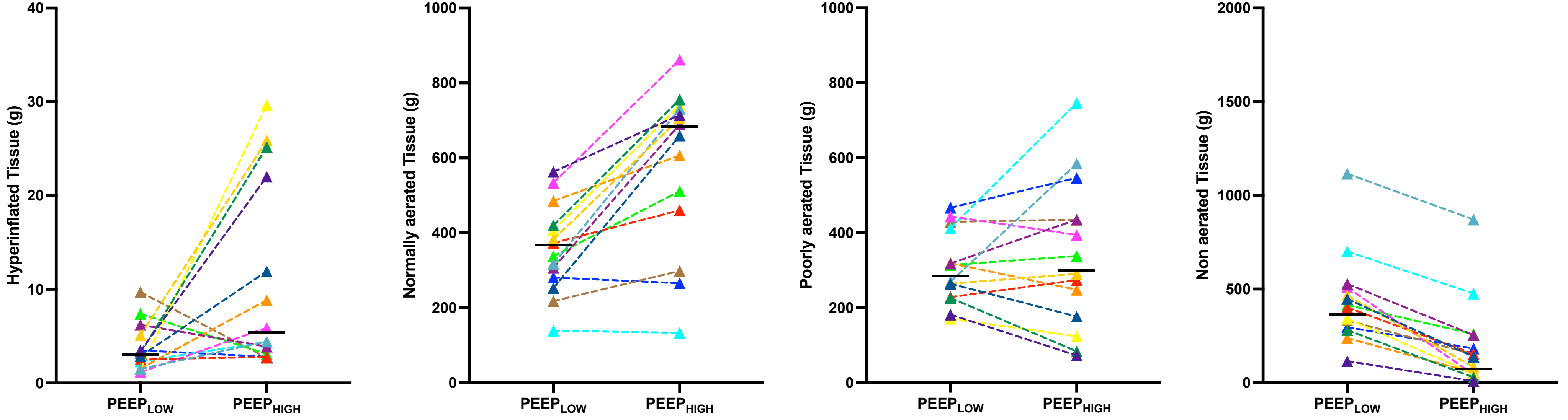

END-EXPIRATION

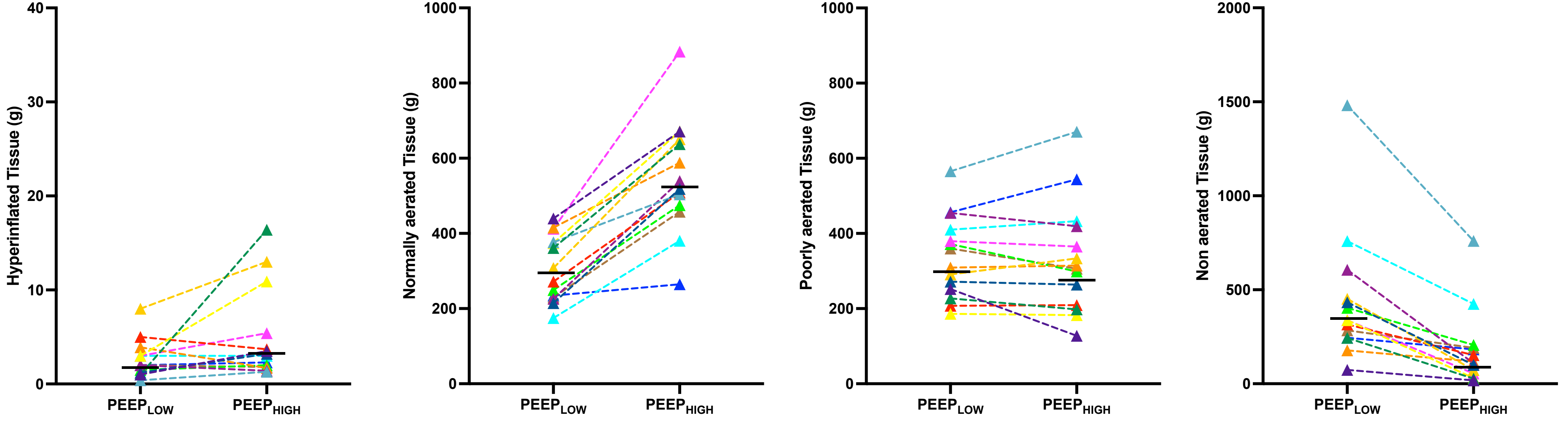

Supplement: Supplementary file 1 — Supplementary Material 1. [file 13613_2024_1343_MOESM1_ESM.pdf]
